# miR-16-5p inhibits chordoma cell proliferation, invasion and metastasis by targeting Smad3

**DOI:** 10.1038/s41419-018-0738-z

**Published:** 2018-06-07

**Authors:** Hongliang Zhang, Kang Yang, Tingting Ren, Yi Huang, Xiaodong Tang, Wei Guo

**Affiliations:** 1 Musculoskeletal Tumor Center, Peking University People’s Hospital, No. 11 Xizhimen South Street, Beijing, 100044 People’s Republic of China; 2 Beijing Key Laboratory of Musculoskeletal Tumor, No. 11 Xizhimen South Street, Beijing, 100044 People’s Republic of China

## Abstract

Aberrantly expressed miRNAs play a crucial role in the development of multiple cancer types, including chordoma. However, the detailed molecular mechanisms are unclear and need to be elucidated. In this study, miRNAs were screened by miRNA array analysis and then confirmed by real-time PCR analysis. We found that miR-16-5p was significantly downregulated in chordoma, and overexpression of miR-16-5p suppressed chordoma cell proliferation, invasion and migration in vitro and in vivo and correlated with the upregulated expression of E-cadherin and downregulated expression of N-cadherin and vimentin. Furthermore, Smad3 was identified as a target of miR-16-5p, and Smad3 was highly expressed in chordoma tissues. Further research showed that knockdown of Smad3 had an effect similar to that of overexpression of miR-16-5p in chordoma cells. Our findings demonstrate that miR-16-5p plays a tumor suppressor role in chordoma progression by targeting Smad3, which could provide a promising prognostic and therapeutic strategy for chordoma treatment.

## Background

Chordoma is a rare mesenchymal tissue tumor that accounts for 1–4% of all bone malignancies^1^. Recent data suggest that these tumors arise from notochord remnants^2^. Although chordoma is considered a comparatively low malignancy, it has a high recurrence rate and can metastasize to nearby tissues^3,4^. Chordoma is resistant to conventional chemotherapy and radiotherapy, which makes surgical resection the most effective treatment for chordoma. However, complete en bloc excision is frequently impossible because of the anatomical location of the tumors, and patients are vulnerable to relapse after surgery^5–7^. Therefore, exploring novel therapeutic targets for patients with chordoma is urgently needed.

MicroRNAs (miRNAs) are a class of highly conserved small non-coding regulatory RNAs that are 17–25 nucleotides in length and that can promote the degradation of messenger RNAs (mRNAs) or inhibit their translation by partial complementary binding, particularly to the 3′-untranslated regions (3′-UTRs) of mRNAs^8^. Many studies show that miRNA dysregulation is important for tumor initiation and progression and can act as either oncogenes or tumor suppressors in different cancers, including chordoma^9–11^. For example, a previous study demonstrated that highly expressed miR-155 independently affects the prognosis of chordoma, while another report showed that miR-1 is downregulated and directly targets the Slug gene in chordoma^12,13^. However, the relevance and significance of the majority of miRNAs in chordoma remain unclear.

In this study, using miRNA array, we compared the expression profile of miRNAs in chordomas to that of nucleus pulposus samples to determine which miRNAs might be involved in the molecular pathogenesis of chordomas. After quantification with real-time reverse transcription PCR (RT-PCR) confirmed the miRNA expression profile among samples, we found that miR-16 was significantly downregulated in chordoma. Functional analyses showed that overexpression of miR-16 inhibited chordoma cell proliferation, invasion and migration. Furthermore, Smad3 was identified as a target of miR-16-5p and was highly expressed in chordoma tissues. Our results show that knockdown of Smad3 had an effect similar to that of overexpression of miR-16-5p in chordoma cells. These findings show that miR-16 functions as a tumor suppressor in chordoma development, which could provide a promising prognostic and therapeutic strategy for chordoma treatment.

## Materials and methods

### Clinical tissue specimen

Twenty-two chordoma tissues and 12 nucleus pulposus tissues were collected under the protocols approved by the Ethics Committee of Peking University People’s Hospital, and informed consent was obtained from all patients. The nucleus pulposus was derived from adult patients who had undergone total sacrectomy due to tumors, and we got nucleus pulposus from the intervertebral disc of L5/S1 which was healthy. The clinical characteristics of these patients are shown in Table 1. Fifty-four paraffin-embedded pathological chordoma specimens were obtained from the Department of Pathology and the Musculoskeletal Tumor Center, Peking University People’s Hospital (Beijing, China).

**Table 1:** Clinical characteristics of nucleus pulposus and patients with chordoma

Characteristics	Chordoma cases	Nucleus pulposus
Gender		
Male	11	8
Female	11	4
Age (years)		
≤60	13	9
>60	9	3
Relapse		
Yes	10	NA
No	12	NA
Sites		
Sacrum	22	NA
Other	0	NA

*NA* not applicable

### Cell culture and reagents

The human chordoma cell lines U-CH1 and U-CH2 were both obtained from American Type Culture Collection (ATCC, Manassas, VA, USA) and were cultured in a 1:4 ratio of Iscove’s modified Dulbecco’s modified Eagle’s medium (Gibco, Grand Island, NY, USA) and RPMI-1640 medium (Gibco) supplemented with 10% fetal bovine serum (FBS; Gibco) and 1% penicillin/streptomycin (Gibco) in a humidified incubator with a 5% CO_2_/95% air atmosphere at 37 °C. Culture flasks were coated with rat tail type I collagen (BD Biosciences, San Diego, CA, USA) prior to use. The following antibodies were used in the experiments: anti-Vimentin, anti-N-cadherin and anti-GAPDH were obtained from Cell Signaling Technology (Beverly, MA, USA), and anti-Smad3 and anti-E-cadherin were obtained from Abcam (USA). Smad3 small interfering RNA (siRNA) was purchased from Suzhou GenePharma (Suzhou, China). Lipofectamine 3000 was purchased from Origene (Rockville, MD, USA).

### Quantitative RT-PCR (qRT-PCR)

The miRNAs were isolated from chordoma tissues or cell lines using an RNeasy/miRNeasy Mini Kit (Qiagen, Limburg, The Netherlands) according to the manufacturer’s instructions. Total RNA was isolated using TRIzol reagent (Invitrogen). The complementary DNAs (cDNAs) were synthesized using a RevertAid^TM^ First-Strand cDNA Synthesis Kit (Fermentas, Vilnius, Lithuania), and real-time quantitative PCR was carried out using SYBR-Green PCR Master Mix (Applied Biosystems, Foster City, CA, USA) on a 7900 Real-Time PCR System (Applied Biosystems). U6 or glyceraldehyde 3-phosphate dehydrogenase (GAPDH) was used as an endogenous control. The primers used in this study are listed in Table 2. All experiments were repeated at least three times.

**Table 2:** Primers for real-time PCR

Primers		Sequences
E-cadherin	F	5′-TGCTCACATTTCCCAACTC-3'
	R	5′-TCTGTCACCTTCAGCCATC-3'
N-cadherin	F	5′-CTGACAATGACCCCACAGC-3'
	R	5′-TCCTGCTCACCACACTACTT-3'
Vimentin	F	5′-CTGGATTTCCTCTTCGTGGA-3'
	R	5′-CGAAAACACCCTGCAATCTT-3'
GAPDH	F	5′-GCACCGTCAAGGCTGAGAAC-3'
	R	5′-ATGGTGGTGAAGACGCCAGT-3'
Smad3	F	5′-GTCTGCAAGATCCCACCAG-3’
	R	5′-AGCCCTGGTTGACCGACT-3’
miR-16-5p	F	5′-TAGCAGCACGTAAATATTGGCG-3'
	R	5′-TGCGTGTCGTGGAGTC-3’
U6	F	5′-CTCGCTTCGGCAGCACA-3'
	R	5′-AACGCTTCACGAATTTGCGT-3'

### Protein extraction and western blot

The indicated cells were lysed with RIPA buffer. Equal amounts of proteins collected from different types of cell lysates were loaded on 10–15% sodium dodecyl sulfate–polyacrylamide gel electrophoresis gels using a NuPAGE system (Invitrogen) and then transferred onto polyvinylidene difluoride membranes. The membranes were blocked with non-fat dry milk at room temperature and then incubated with primary antibodies at 4 °C overnight. Membranes were then washed and incubated with secondary antibodies. Proteins were visualized by electrochemiluminescence western blot substrate detection (Pierce). All experiments were repeated at least three times.

### Cell transfection

siSmad3, scrambled negative control siRNA, miR-16-5p mimics and negative control were obtained from GenePharma (Suzhou, Jiangsu, China). Chordoma cells were transfected with siRNA, miR-16-5p mimics and their negative control by Lipofectamine 3000 (Invitrogen) according to the manufacturer’s instructions. The final concentration of miRNA mimics was 100 nM, and the final concentration of siRNA was 20 nM.

The sequence of mature miR for miR-16-5p is 5′-UAGCAGCACGUAAAUAUUGGCG-3′, and the negative control is 5′-UUCUCCGAACGUGUCACGUTT-3′. The sense sequence of Smad3 siRNA is 5′-CCGCAUGAGCUUCGUCAAATT-3′, and the antisense sequence is 5′-UUUGACGAAGCUCAUGCGGTT-3′. The negative control siRNA sequences are 5′-UUCUCCGAACGUGUCACGUTT-3′ and 5′-ACGUGACACGUUCGGAGAATT-3′.

### CCK-8 assay

U-CH1 and U-CH2 cells were plated in 96-well plates at a density of 5000 cells in 100 μl medium per well in triplicate. Then, the cells were transfected with 50 nM miR-16-5p mimics and negative control (Suzhou GenePharma Co., Ltd.) using Lipofectamine 3000. Cell viability was examined daily for 4 days using CCK-8 (Dojindo Laboratories, Kumamoto, Japan) according to the manufacturer’s instructions.

### Wound-healing assay

Transfected U-CH1 and U-CH2 cells and their negative control were seeded in a 6-well culture plate (5 × 10^5^ cells) and cultured to a confluent state. An artificial wound was introduced with a P-200 pipette tip in each well. The data of the wounded area were recorded at 0 h and 48 h with a microscope. Three replicates of each condition were used.

### Transwell assay

Briefly, transfected U-CH1 and U-CH2 cells and their negative control were resuspended in serum-free medium. Then, 200 mL (5 × 10^4^ cells) was seeded in the upper chambers of migration or invasion chambers (BD Biosciences). The bottom chamber was filled with 600 mL culture medium with 20% FBS. After 48 h, the cells in the upper chamber were removed with a swab, and the cells that migrated to the lower layer and attached to the membrane were stained with 0.1% crystal violet and were counted in five fields per well under a microscope. Experiments were repeated three times.

### Immunohistochemistry

Paraffin sections were reacted with antibodies (1:100 dilution) and then stained with a rabbit serum instead of target antibody as a negative control. Cells exhibiting positive staining on cell membranes and in the cytoplasm and nucleus were counted in at least 10 representative fields (×400 magnification), and the mean percentage of positive cells was calculated. Immunostaining was evaluated by two independent pathologists blinded to clinical information. Specimens were scored according to the intensity of the dye color and the number of positive cells. The intensity of the dye color was graded as 0 (no color), 1 (light yellow), 2 (light brown) or 3 (brown), and the number of positive cells was graded as 0 (<5%), 1 (5–25%), 2 (25–50%), 3 (51–75%) or 4 (>75%). The two grades were added together and specimens were assigned to one of 4 levels: 0–1 score (−), 2 scores (+), 3–4 scores (++) and more than 5 scores (+++). The positive expression rate was expressed as the percent of the addition of (++) and (+++) to the total number.

### Luciferase reporter assay

The Smad3 3′-UTR containing the wild-type or mutated miR-16-5p binding sequences was synthesized by Genscript (Nanjing, Jiangsu, China) and cloned into the pmirGLO luciferase reporter vector (Promega, Madison, WI, USA). U-CH1 cells were transfected with the wild-type/mutant Smad3 luciferase reporter vector and miR-16-5p mimic or negative control using Lipofectamine 3000. Luciferase activity was measured using a Dual-Luciferase Reporter Assay System (E191, Promega), and the results are expressed as firefly luciferase activity normalized to *Renilla* luciferase activity. Each sample was measured in triplicate, and the experiment was repeated at least three times.

### Microarray array analysis

Total RNA was extracted from 12 chordoma tissues and 12 nucleus pulposus tissues using an RNeasy Mini Kit (Qiagen, Venlo, The Netherlands) and reverse transcribed according to the manufacturer’s instructions (Fermentas, Waltham, MA, USA). Microarray chip analysis was performed and analyzed by a commercial company (Phalanx Biotech Group, Hsinchu, Taiwan) using the Human v7.1 miRNA OneArray Platform. The threshold for differentially expressed genes was log_2_ | Fold change| ≧ 0.585 and *p* value < 0.05.

### Tumor xenografts

The 6-week-old BALB/c athymic nude mice (Vitalriver, Beijing, China) were subcutaneously injected in the right flank with 5 × 10^6^ U-CH1 cells. The mice were fed under specific pathogen-free conditions, and when a palpable mass developed, they were randomly divided into two groups. Then, 10 nmol hsa-mir-16-5p agomir (Ribobio Co. Guangzhou, China) for in vivo RNA delivery or negative control in 0.1 ml saline buffer was locally injected into the tumor mass once every week for 8 weeks. The tumor volume (length × width^2^/2) was measured every week, and the mice were killed after 8 weeks. Tumor samples were processed for routine qRT-PCR and immunohistochemistry (IHC).

### Statistical analyses

SPSS 21.0 software (Chicago, IL, USA) was used for statistical analyses. The data were analyzed by Student’s *t*-test or one-way ANOVA, and the results are presented as the mean ± S.D. Significant data are indicated by *p < 0.05, **p < 0.01 and ***p < 0.001.

## Results

### Identification of miRNAs differentially expressed in chordoma samples

**Fig. 1:**
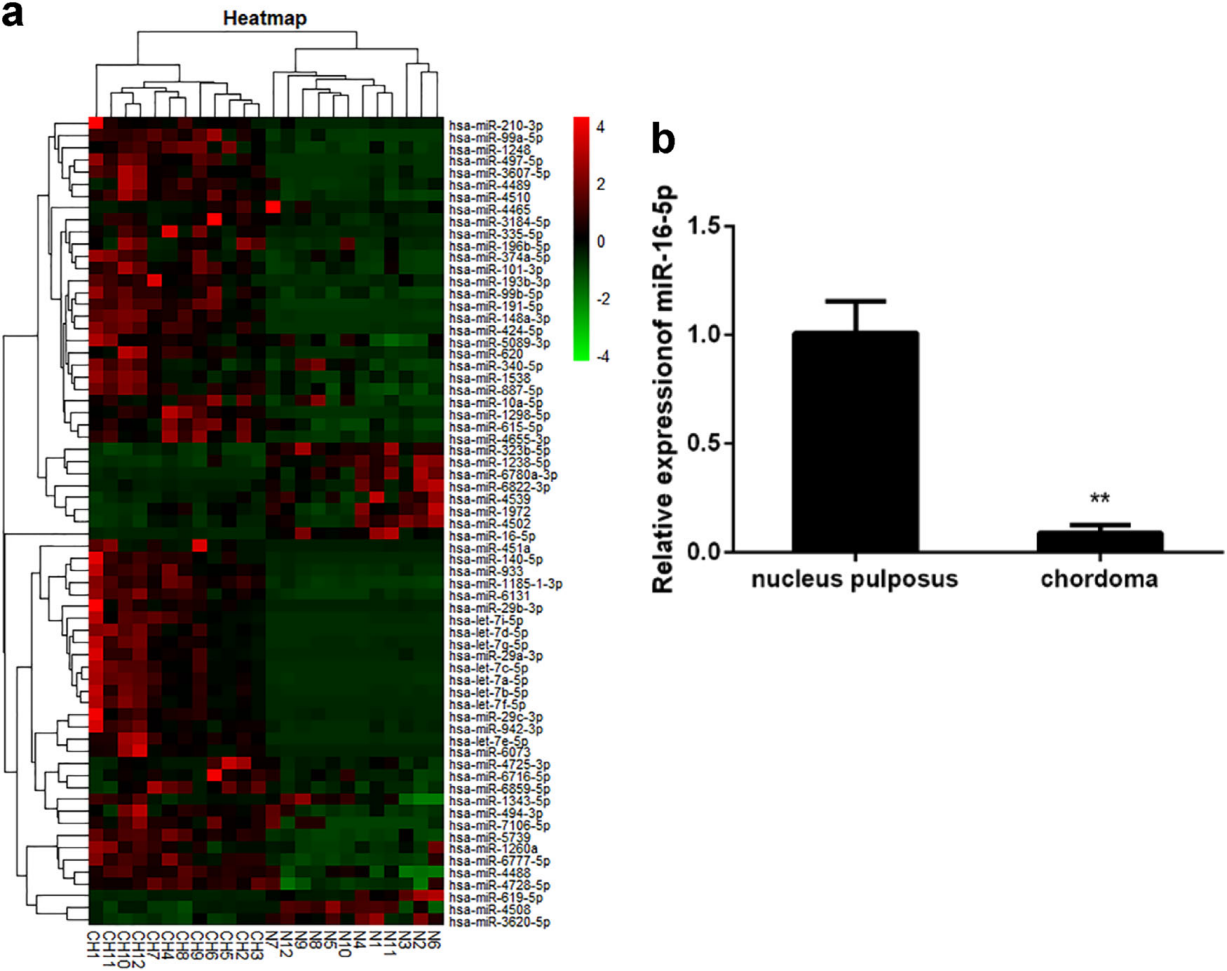
The miR-16-5p expression level was downregulated in chordoma tissues. **a** Heat-map representation of the differentially expressed miRNA patterns in chordoma (CH) versus nucleus pulposus tissues (N). Relative expression is presented as a colorgram (red: high expression). **b** Expression of miR-16-5p as determined by real-time RT-PCR analysis. A comparison of the average relative expression levels of miR-16-5p in chordoma and nucleus pulposus samples is shown. **p < 0.01.

To determine whether there are differences in the levels of miRNA expression between chordoma and nucleus pulposus tissues, we analyzed miRNAs from 12 chordoma samples and compared them with miRNAs from 12 nucleus pulposus tissue samples. Using miRNA array, we identified 126 miRNAs that were dysregulated significantly in chordoma tissues when compared with their expression in nucleus pulposus (*p* < 0.05). Among these miRNAs, 102 were upregulated, and 24 were downregulated (Fig. 1). Then, 27 miRNAs were chosen for further validation. In the validation set, the concentration of miR-16 was measured by qRT-PCR in a group comprising 10 chordoma tissue samples and 5 nucleus pulposus samples. miR-16-5p was significantly downregulated in chordoma tissue samples compared with control samples, consistent with the miRNA array results. Thus, we focused on miR-16-5p for further study.

### miR-16-5p suppressed cell proliferation in vitro

**Fig. 2:**
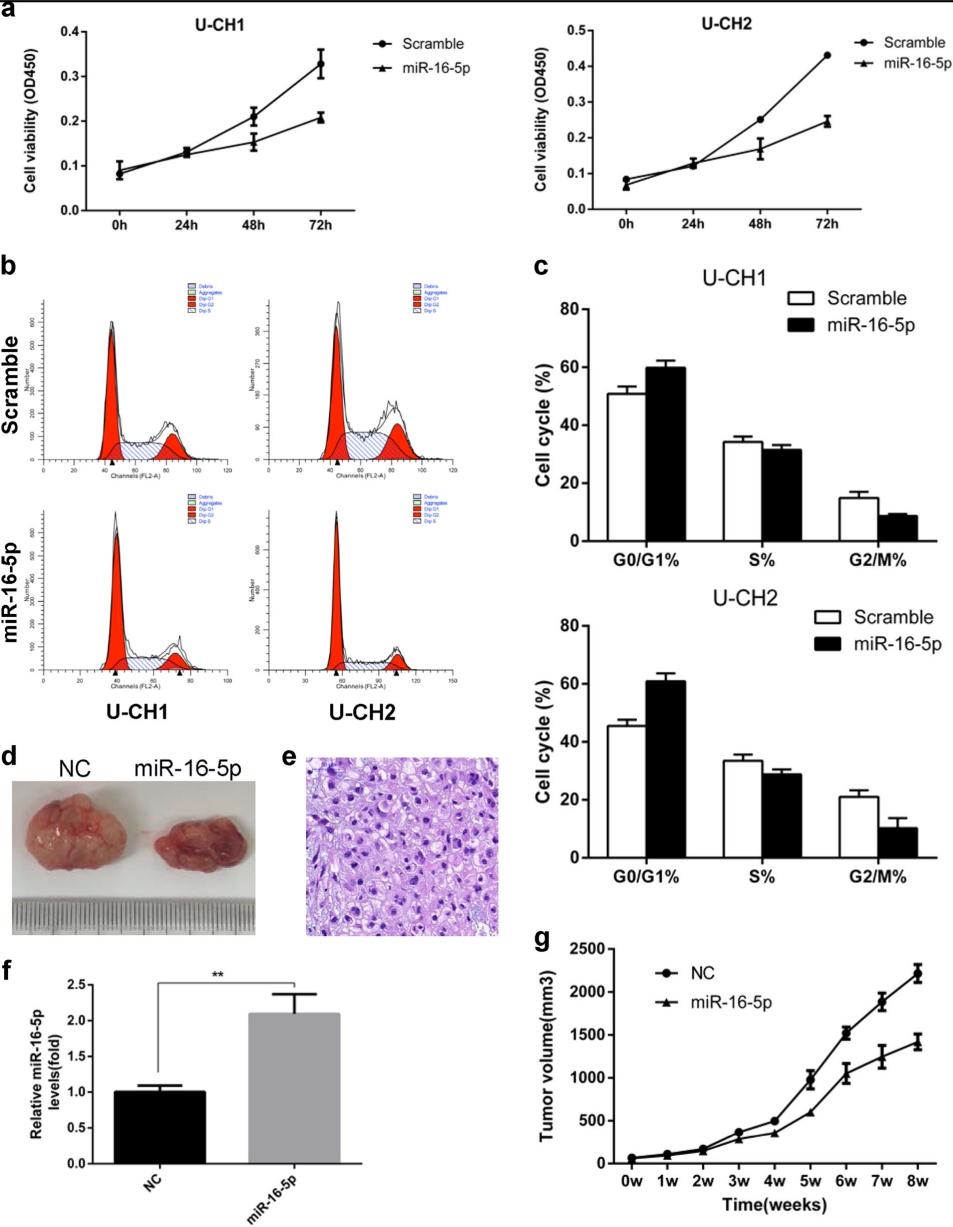
miR-16-5p suppressed cell proliferation in vitro and vivo. **a** Cell proliferation was evaluated by the CCK-8 assay at 24, 48 and 72 h. **b** The effects of miR-16-5p on the cell cycle were determined by flow cytometry (FCM). **c** FCM indicated G0/G1-phase arrest of the cell cycle in cells transfected with miR-16-5p mimics. **d** Tumor volume was significantly decreased in the miR-16-5p agomir treatment group compared with the control group. **e** Representative images of H&E staining of tumors formed by U-CH1 cells. **f** qRT-PCR results showing that miR-16-5p was significantly upregulated in the miR-16-5p agomir treatment group compared with the control group. **g** The tumor volume was measured every 7 days, and the growth curves of the tumors were plotted accordingly. **p < 0.01.

To identify the function of miR-16-5p in chordoma, U-CH1 and U-CH2 cell lines were transfected with miR-16-5p mimics. Further, CCK-8 assays were performed to measure the effect of miR-16-5p on cell proliferation, and significantly suppressed cell viability was observed in U-CH1 and U-CH2 cells transfected with miR-16-5p mimics compared with negative control (Fig. 2a). In addition, flow cytometry (FCM) indicated similar G0/G1-phase arrest of the cell cycle in cells transfected with miR-16-5p mimics (Fig. 2b). These results collectively suggest that miR-16-5p inhibits cell proliferation.

### The effect of miR-16-5p on tumorigenesis in a xenograft model

To directly investigate the role of miR-16-5p in tumor formation and growth in vivo, U-CH1 cells were subcutaneously implanted into 6-week-old nude mice to form a xenograft model. Then, we injected 10 nmol miR-16-5p agomir or agomir negative control into the tumor mass once a week. The tumor volume was monitored every 7 days, and the growth curves of the tumors were plotted accordingly (Fig. 2g). Finally, the size of the tumor nodules was examined. We found that the tumor volume was significantly decreased in the miR-16-5p agomir treatment group compared with the control group (Fig. 2d). Tumor samples were processed for routine qRT-PCR and western blot, and the results showed that miR-16-5p was significantly upregulated in the miR-16-5p agomir treatment group compared with the control group (Fig. 2f). Hematoxylin and eosin (H&E) staining of the tumor samples was then performed (Fig. 2e). These results suggest that miR-16-5p may act as a suppressor of chordoma proliferation.

### miR-16-5p can suppress chordoma cell invasion and migration

**Fig. 3:**
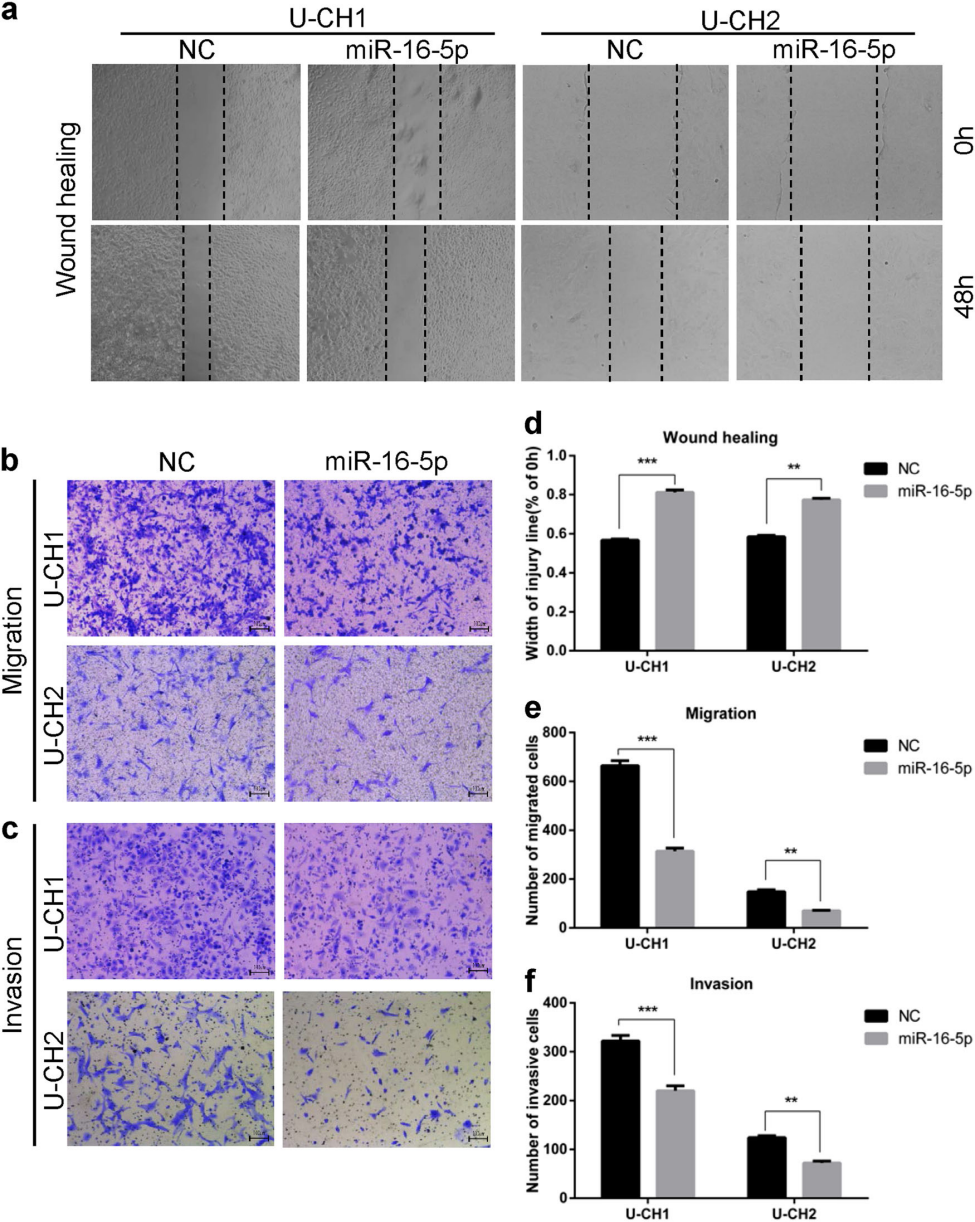
miR-16-5p suppressed the invasion and migration of chordoma cells. **a**–**c** Wound-healing and Transwell migration assays were performed in U-CH1 and U-CH2 cells transfected with miR-16-5p mimics or mimic controls. **d**–**f** Statistical results of wound-healing and Transwell migration assays are shown accordingly. **p < 0.01, ***p < 0.001.

To further study whether the migration and invasion ability of chordoma cells was affected by miR-16-5p, wound-healing and Transwell assays were performed. The results indicated that miR-16-5p mimics can significantly inhibit the migration and invasion of U-CH1 and U-CH2 cells (Fig. 3b). In addition, wound-healing assay results showed that miR-16-5p mimics inhibit the migration potential of U-CH1 and U-CH2 cells (Fig. 3a), a result consistent with the findings above. Together, miR-16-5p can suppress the invasion and migration of chordoma cells.

### miR-16-5p regulates E-cadherin, N-cadherin and vimentin expression

**Fig. 4:**
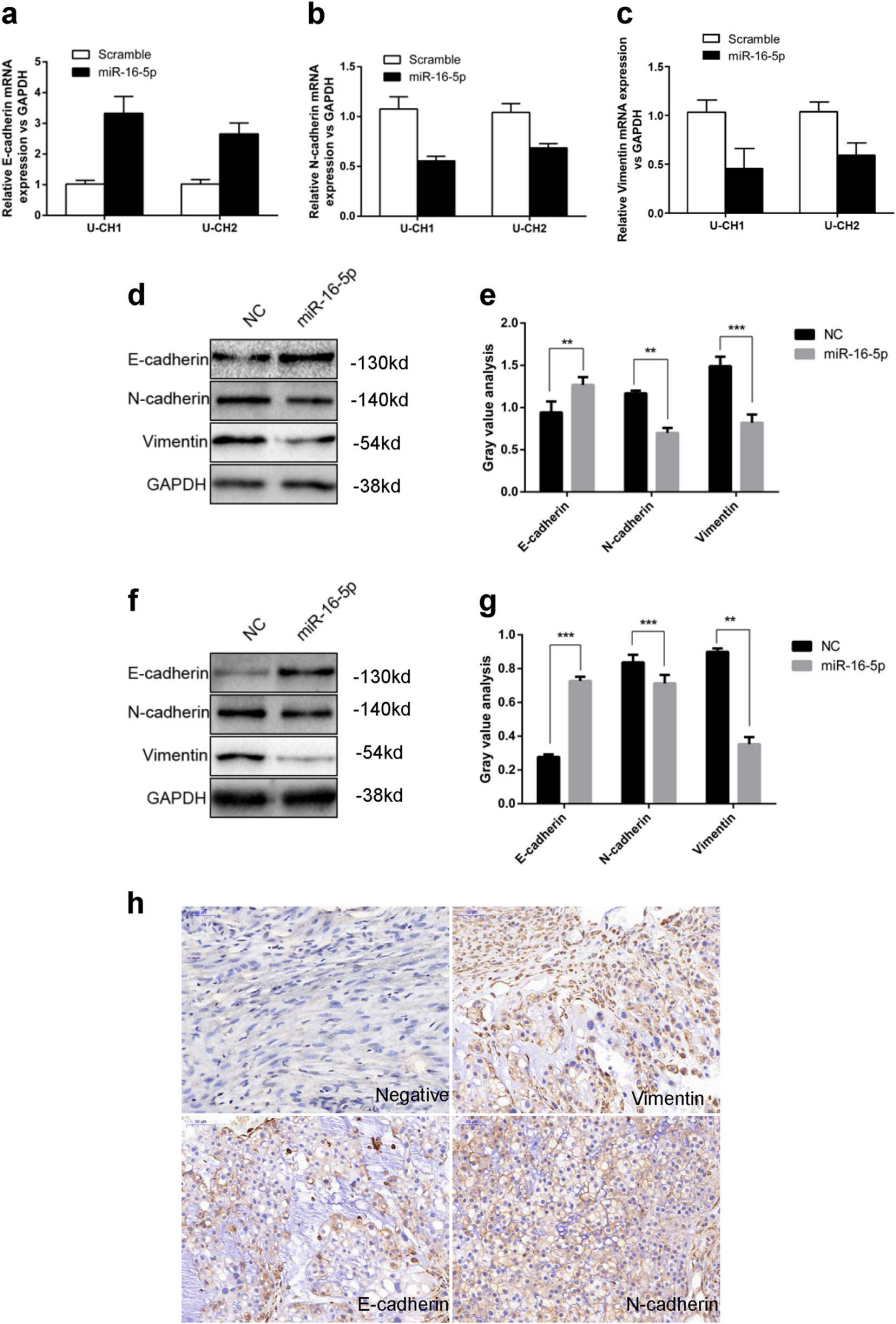
miR-16-5p regulates E-cadherin, N-cadherin and vimentin expression. **a**–**c** mRNA expression levels of E-cadherin, N-cadherin and vimentin in U-CH1 and U-CH2 cells transfected with miR-16-5p mimics or mimic controls were measured by qRT-PCR separately. **d**, **e** The protein expression levels of E-cadherin, N-cadherin and vimentin in U-CH1 cells transfected with miR-16-5p mimics or mimic controls were measured by western blotting. **f**, **g** The protein expression levels of E-cadherin, N-cadherin and vimentin in U-CH2 cells transfected with miR-16-5p mimic or mimic control were measured by western blotting. **h** Representative IHC staining images of EMT markers in chordoma tissues. **p < 0.01, ***p < 0.001.

We performed qRT-PCR and western blotting to further investigate whether miR-16-5p expression influences the protein expression levels of E-cadherin, N-cadherin and vimentin. As shown in Fig. 4, overexpression of miR-16-5p significantly upregulated the expression of E-cadherin and downregulated the expression of N-cadherin and vimentin at both the mRNA and protein levels in U-CH1 and U-CH2 cells.

### The expression of EMT markers in chordoma tissues

We performed IHC in 54 paraffin-embedded pathological chordoma specimens to identify the expression of epithelial–mesenchymal transition (EMT) markers in chordoma tissues (Fig. 4h). The association between Smad3 expression and clinicopathological characteristics was statistically analyzed, and the results revealed that low E-cadherin expression was correlated with surrounding invasion (*p* < 0.05, Table 3); however, there is no significant correlation between clinicopathological characteristics and the expression of N-cadherin and vimentin.

**Table 3:** The expression of EMT markers in chordoma tissues

Biological characteristics	*n*	E-cadherin				N-cadherin				Vimentin			
		Positive	Negative	*χ* ^2^	*p*	Positive	Negative	*χ* ^2^	*p*	Positive	Negative	*χ* ^2^	*p*
Age (years)													
>60	24	10	14	0.14	0.783	17	7	0.236	0.627	21	3	1.634	0.201
≤60	30	11	19			23	7			29	1		
Gender													
Male	38	12	26	2.884	0.128	30	8	1.586	0.208	36	2	0.86	0.354
Female	16	9	7			10	6			14	2		
Relapse													
Yes	14	8	6	2.65	0.104	9	5	0.943	0.332	14	0	1.512	0.219
No	40	13	27			31	9			36	4		
Surrounding invasion													
Yes	36	10	26	5.61	0.018	29	7	2.363	0.124	33	3	0.135	0.713
No	18	11	7			11	7			17	1		
Enneking stage													
IA	2	1	1	1.174	0.556	2	0	1.929	0.381	2	0	3.218	0.2
IB	49	18	31			35	14			46	3		
III	3	2	1			3	0			2	1		

### Validation of Smad3 as a direct downstream target of miR-16-5p

**Fig. 5:**
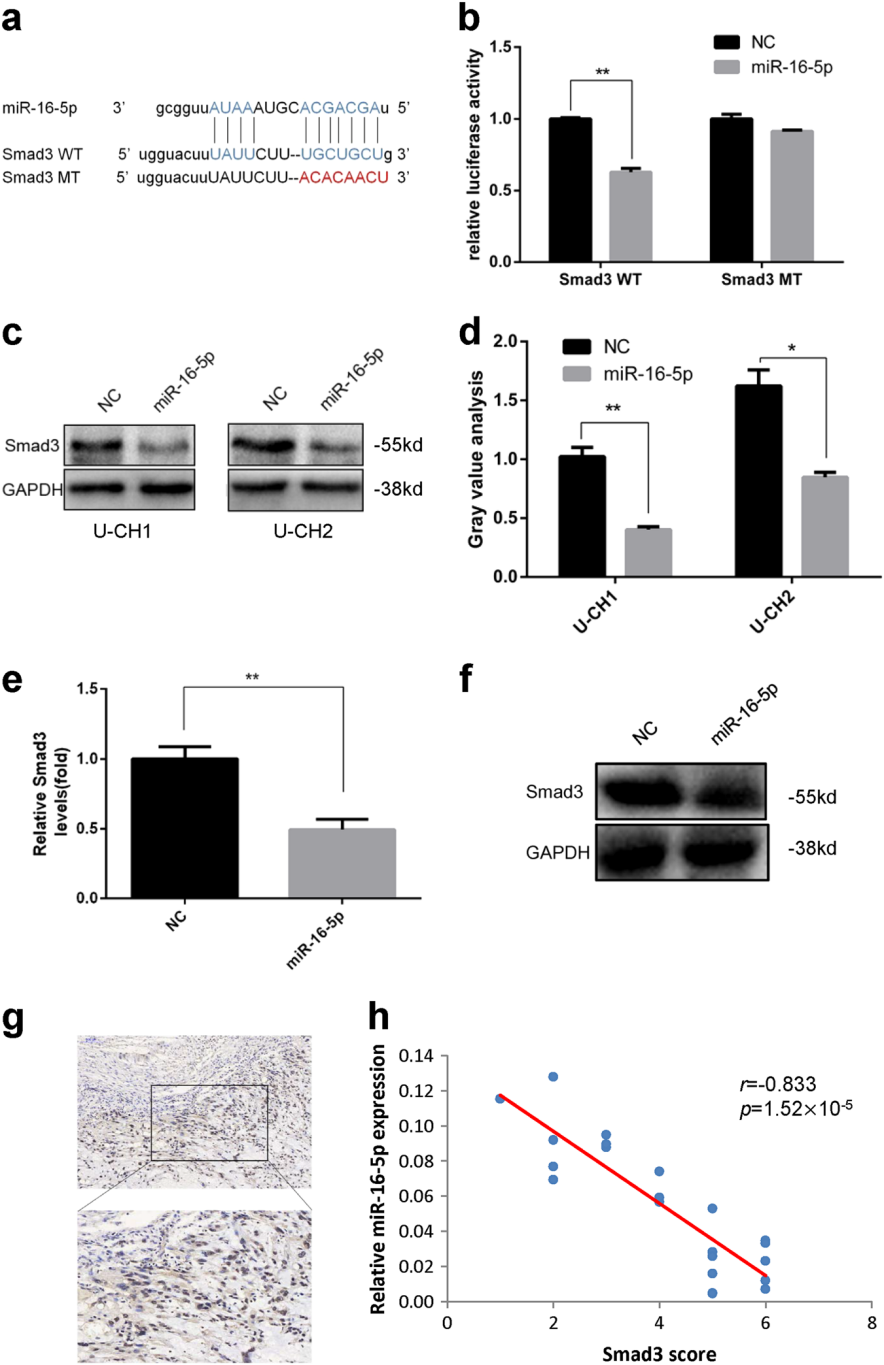
Identification of Smad3 as a target of miR-16-5p. **a** Schematic representation of Smad3 3′-UTR containing the wild-type or mutant binding site for miR-16-5p. **b** Luciferase reporter activity following expression after transfection (mimic control and miR-16-5p mimic) in U-CH1 cells. **c** Western blot analysis of Smad3 expression in miR-125b-overexpressed cells. **d** Gray value analysis of western blotting. **e** Smad3 expression level in xenograft tissue by real-time PCR. **f** Smad3 expression level in xenograft tissue by western blot. **g** Representative IHC staining images of Smad3 expression in chordoma tissues. **h** High Smad3 expression was correlated with low miR-16-5p expression. *p < 0.05, **p < 0.01.

To investigate the mechanism by which miR-16-5p affects chordoma cells, we used bioinformatics tools (TargetScan, miRanda and PicTar) to predict its potential target genes, and Smad3 was identified as a likely target of miR-16-5p because there was complementarity between miR-16-5p and the Smad3 3′-UTR (Fig. 5a). Then, we performed a luciferase reporter assay to confirm that miR-16-5p directly binds to the 3′-UTR of Smad3 in chordoma cells. Our results showed that overexpression of miR-16-5p significantly reduced luciferase activity of the reporter gene in wild type, but not mutant, indicating that miR-16-5p directly targeted the Smad3 3′-UTR (Fig. 5b). Then, we further confirmed the effect by western blot. As shown in Fig. 5c, the expression of Smad3 was significantly downregulated in miR-16-5p overexpressing chordoma cells. Furthermore, we measured Smad3 expression level in xenograft tissue using both real-time PCR and western blot, and the expression of Smad3 was significantly downregulated in miR-16-5p overexpressed xenograft tissue (Fig. 5e, f). Taken together, our results demonstrate that Smad3 is a direct target of miR-16-5p in chordoma cells and that miR-16-5p directly regulates Smad3 expression at the posttranscriptional level.

### Smad3 was highly expressed in chordoma tissues

To identify the expression of Smad3 in chordoma tissues, we performed IHC in 54 paraffin-embedded pathological chordoma specimens and found that Smad3 was highly expressed in chordoma tissues (Fig. 5g). The association between Smad3 expression and clinicopathological characteristics was statistically analyzed, and the results revealed that high Smad3 expression was correlated with surrounding invasion (*p* < 0.05, Table 4). The association between Smad3 expression and miR-16-5p expression was statistically analyzed, and the results revealed that high Smad3 expression was correlated with low miR-16-5p expression (Fig. 5h).

**Table 4:** The expression of Smad3 in chordoma tissues

Biological characteristics	*n*	Positive	Negative	*χ* ^2^	*p*
Age (years)					
>60	24	16	8	0	1
≤60	30	20	10		
Gender					
Male	38	26	12	0.178	0.673
Female	16	10	6		
Relapse					
Yes	14	10	4	0.193	0.661
No	40	26	14		
Surrounding invasion					
Yes	36	28	8	6	0.014
No	18	8	10		
Enneking stage					
IA	2	1	1	0.26	0.878
IB	49	33	16		
III	3	2	1		

### Knockdown of Smad3 has an effect similar to that of overexpression of miR-16-5p in chordoma cells

**Fig. 6:**
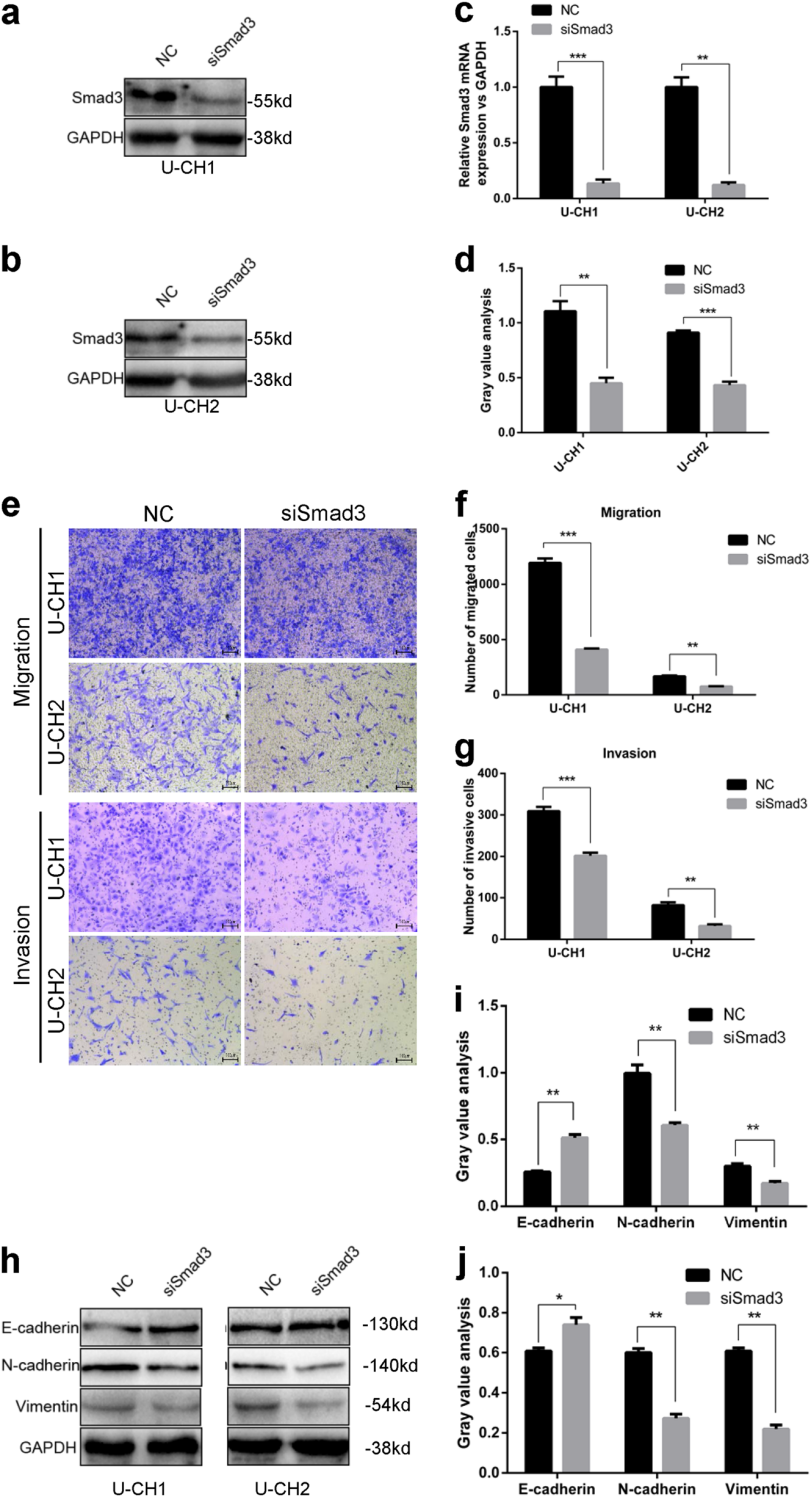
Knockdown of Smad3 had a similar effect as overexpression of miR-16-5p in chordoma cells. **a**–**c** Smad3 siRNA was transfected into U-CH1 and U-CH2 cells. Downregulated Smad3 was confirmed by western blotting and qRT-PCR; gray value analysis of western blotting is shown in (**d**). **e**–**g** Transwell assay results showed that knockdown of Smad3 significantly suppressed migration and invasion of U-CH1 and U-CH2 cells. **h** Western blotting results showed that knockdown of Smad3 significantly upregulated the expression of E-cadherin and downregulated the expression of N-cadherin and vimentin in U-CH1 and U-CH2 cells. Gray value analysis of western blotting in U-CH1 (**i**) and U-CH2 (**j**) cells is shown. *p < 0.05, **p < 0.01, ***p < 0.001.

To further investigate the role of Smad3 in chordoma cells, we transfected Smad3 siRNA into U-CH1 and U-CH2 cells and then confirmed the downregulation of Smad3 by qRT-PCR and western blotting (Fig. 6a–d). As illustrated in Fig. 6e, the knockdown of Smad3 significantly suppressed the migration and invasion of U-CH1 and U-CH2 cells (Fig. 6e–g). Using western blotting, we further investigated whether the expression of E-cadherin, N-cadherin and vimentin could be influenced by Smad3. As shown in Fig. 6h, knockdown of Smad3 significantly upregulated the expression of E-cadherin and downregulated the expression of N-cadherin and vimentin in U-CH1 and U-CH2 cells, which had an effect similar to that of overexpression of miR-16-5p.

## Discussion

Chordoma is a rare mesenchymal tissue tumor with low malignancy. It has a high recurrence rate and frequently leads to local invasion and distant metastasis during advanced stages^4^. Eradicating by surgery is difficult due to the complicated anatomical location of the tumors, and patients are vulnerable to relapse after surgery. Furthermore, chordoma is resistant to conventional chemotherapy and radiotherapy, which makes researching the detailed molecular mechanisms underlying chordoma progression and exploring novel therapeutic targets for patients urgently needed^5–7^.

However, because it is rare and research tools are very limited, few molecular and functional studies of chordoma have been published^14^. Currently, accumulating evidence has shown that aberrantly expressed miRNAs play a crucial role in the development of multiple cancer types, including chordoma^15–17^. Several studies have indicated that aberrantly expressed miRNAs may influence the progression of human chordoma. It has been reported that miR-1, miR-31 and miR-663a potentially act as tumor-suppressive miRNAs in chordoma^13,18,19^. However, the detailed molecular mechanisms have yet to be elucidated.

We performed miRNA array analysis to screen for differentially expressed miRNAs in chordoma samples and found that miR-16-5p was significantly downregulated in chordoma samples compared with that in nucleus pulposus samples, which means that miR-16-5p may act as a tumor suppressor in chordoma. To research the specific function of miR-16-5p in chordoma, we overexpressed miR-16-5p in chordoma cells and found that cell proliferation, invasion and migration were suppressed significantly and correlated with the upregulated expression of E-cadherin and downregulated expression of N-cadherin and vimentin. Then, using U-CH1 cell lines, we constructed a xenograft model of human chordoma cells in nude mice, which are rarely used in chordoma research, and found that overexpression of miR-16-5p can suppress tumor growth in vivo.

To explore the mechanism by which miR-16-5p affects chordoma cells, bioinformatics tools were used, and Smad3 was identified as a potential target of miR-16-5p. Then, using a luciferase reporter assay, we confirmed that Smad3 was a direct target of miR-16-5p in chordoma cells and that miR-16-5p directly regulated Smad3 expression at the posttranscriptional level. Furthermore, Smad3 was highly expressed in chordoma tissues and it was correlated with surrounding invasion, and further research showed that knockdown of Smad3 had the same effect as overexpression of miR-16-5p in chordoma cells. Taken together, our findings demonstrate a tumor suppressor role of miR-16-5p in chordoma progression by targeting Smad3, which could provide a promising prognostic and therapeutic strategy for chordoma treatment.

As a highly conserved miRNA, miR-16 is frequently deleted or downregulated in many types of cancer, including chronic lymphocytic leukemia, prostate cancer, hepatocellular carcinoma, breast cancer, ovarian cancer, non-small cell lung cancer, gastric cancer, pituitary adenoma and multiple myeloma^20–28^. Thus, miR-16 is generally thought to be a key tumor-suppressive miRNA, and many studies have shown that miR-16 can modulate the cell cycle, inhibit cell proliferation, attenuate cell invasion, promote cell apoptosis and suppress tumorigenesis by targeting B-cell lymphoma 2 (Bcl-2), CCND1 (cyclin D1), CCND3 (cyclin D3), CCNE1 (cyclin E1), CDK6 (cyclin-dependent kinase 6) and WNT3A (wingless-type MMTV integration site family, member 3A) in various cancers^21, 29–31^. However, to date, there has been no report of its role in chordoma, and our research is the first to demonstrate the tumor suppressor role of miR-16-5p in chordoma.

As a major intracellular mediator in the transforming growth factor-β signaling pathway, Smad3 plays an important role in the progression of many cancers^32^. For example, Smad3 is downregulated in glioblastoma tumors and acts as a proliferation inhibitor^33^. Currently, an increasing number of reports show that Smad3 can promote invasion and metastasis by EMT in various cancers, such as lung adenocarcinoma, prostate cancer and pancreatic ductal adenocarcinoma^34–36^. Our results showed that the expression of Smad3 was upregulated in chordoma compared with that in muscle. Knockdown of Smad3 suppressed the migration and invasion of chordoma cells and was accompanied by the upregulation of E-cadherin and downregulation of N-cadherin and vimentin, which indicated that Smad3 may be involved in the metastasis of late-stage chordoma by promoting EMT.

## Conclusion

Our findings demonstrated a tumor suppressor role of miR-16-5p in chordoma progression by targeting Smad3, which provides new insight into the molecular mechanism of chordoma and may offer a possible therapeutic strategy for chordoma treatment. However, more research is needed to understand the exact molecular mechanism of chordoma.

### Data availability

All data generated or analyzed during this study are included in this published article.

## References

[1] Almefty K, Pravdenkova S, Colli BO, Al-Mefty O, Gokden M (2007). Chordoma and chondrosarcoma: similar, but quite different, skull base tumors. Cancer.

[2] Kreshak J (2014). Difficulty distinguishing benign notochordal cell tumor from chordoma further suggests a link between them. Cancer Imaging.

[3] Osaka S (2006). Clinical significance of a wide excision policy for sacrococcygeal chordoma. J. Cancer Res. Clin. Oncol..

[4] Catton C (1996). Chordoma: long-term follow-up after radical photon irradiation. Radiother. Oncol..

[5] York JE (1999). Sacral chordoma: 40-year experience at a major cancer center. Neurosurgery.

[6] Hsieh PC (2009). Long-term clinical outcomes following en bloc resections for sacral chordomas and chondrosarcomas: a series of twenty consecutive patients. Spine (Phila. Pa 1976).

[7] Fuchs B, Dickey ID, Yaszemski MJ, Inwards CY, Sim FH (2005). Operative management of sacral chordoma. J. Bone Joint Surg. Am..

[8] Medina PP, Slack FJ (2008). microRNAs and cancer: an overview. Cell Cycle.

[9] Yu SL (2008). MicroRNA signature predicts survival and relapse in lung cancer. Cancer Cell.

[10] Ng EK (2009). Differential expression of microRNAs in plasma of patients with colorectal cancer: a potential marker for colorectal cancer screening. Gut.

[11] Duan Z (2010). Differential expression of microRNA (miRNA) in chordoma reveals a role for miRNA-1 in Met expression. J. Orthop. Res..

[12] Osaka E (2015). MicroRNA-155 expression is independently predictive of outcome in chordoma. Oncotarget.

[13] Osaka E (2014). MicroRNA-1 (miR-1) inhibits chordoma cell migration and invasion by targeting slug. J. Orthop. Res..

[14] Zhang Y, Schiff D, Park D, Abounader R (2014). MicroRNA-608 and microRNA-34a regulate chordoma malignancy by targeting EGFR, Bcl-xL and MET. PLoS One.

[15] Ma F (2017). MiR-361-5p inhibits glycolytic metabolism, proliferation and invasion of breast cancer by targeting FGFR1 and MMP-1. J. Exp. Clin. Cancer Res..

[16] Ries J (2014). miR-186, miR-3651 and miR-494: potential biomarkers for oral squamous cell carcinoma extracted from whole blood. Oncol. Rep..

[17] Duan Z (2014). Prognostic significance of miRNA-1 (miR-1) expression in patients with chordoma. J. Orthop. Res..

[18] Bayrak OF (2013). MicroRNA expression profiling reveals the potential function of microRNA-31 in chordomas. J. Neurooncol..

[19] Long C (2013). Integrated miRNA-mRNA analysis revealing the potential roles of miRNAs in chordomas. PLoS One.

[20] Pekarsky Y, Croce CM (2015). Role of miR-15/16 in CLL. Cell Death Differ..

[21] Bonci D (2008). The miR-15a-miR-16-1 cluster controls prostate cancer by targeting multiple oncogenic activities. Nat. Med..

[22] Guo CJ, Pan Q, Li DG, Sun H, Liu BW (2009). miR-15b and miR-16 are implicated in activation of the rat hepatic stellate cell: an essential role for apoptosis. J. Hepatol..

[23] Xu F (2010). Loss of repression of HuR translation by miR-16 may be responsible for the elevation of HuR in human breast carcinoma. J. Cell. Biochem..

[24] Bhattacharya R (2009). MiR-15a and MiR-16 control Bmi-1 expression in ovarian cancer. Cancer Res..

[25] Bandi N (2009). miR-15a and miR-16 are implicated in cell cycle regulation in a Rb-dependent manner and are frequently deleted or down-regulated in non-small cell lung cancer. Cancer Res..

[26] Xia L (2008). miR-15b and miR-16 modulate multidrug resistance by targeting BCL2 in human gastric cancer cells. Int. J. Cancer.

[27] Bottoni A (2005). miR-15a and miR-16-1 down-regulation in pituitary adenomas. J. Cell. Physiol..

[28] Corthals SL (2010). Micro-RNA-15a and micro-RNA-16 expression and chromosome 13 deletions in multiple myeloma. Leuk. Res..

[29] Cai CK (2012). miR-15a and miR-16-1 downregulate CCND1 and induce apoptosis and cell cycle arrest in osteosarcoma. Oncol. Rep..

[30] Cimmino A (2005). miR-15 and miR-16 induce apoptosis by targeting BCL2. Proc. Natl. Acad. Sci. USA.

[31] Shi L (2014). p53-induced miR-15a/16-1 and AP4 form a double-negative feedback loop to regulate epithelial-mesenchymal transition and metastasis in colorectal cancer. Cancer Res..

[32] Vincent T (2009). A SNAIL1-SMAD3/4 transcriptional repressor complex promotes TGF-beta mediated epithelial-mesenchymal transition. Nat. Cell Biol..

[33] Wu ZB (2013). The miR-92b functions as a potential oncogene by targeting on Smad3 in glioblastomas. Brain Res..

[34] Yang Y (2013). Targeting Smad2 and Smad3 by miR-136 suppresses metastasis-associated traits of lung adenocarcinoma cells. Oncol. Res..

[35] Thakur N (2014). TGFbeta-induced invasion of prostate cancer cells is promoted by c-Jun-dependent transcriptional activation of Snail1. Cell Cycle.

[36] Yamazaki K (2014). Upregulated SMAD3 promotes epithelial-mesenchymal transition and predicts poor prognosis in pancreatic ductal adenocarcinoma. Lab. Invest..

